# A cross-sectional study exploring mental health literacy among non-athlete personnel in elite-level road cycling

**DOI:** 10.1007/s44192-026-00483-8

**Published:** 2026-06-01

**Authors:** Alexander Smith, Juan Graña, Norman Bitterlich, Jill Colangelo, Xavier Bigard, Ana Buadze, Michael Liebrenz

**Affiliations:** 1 Department of Forensic Psychiatry, University of Bern, Bern, Switzerland; 2 Faculty of Sport, Technology, and Health Sciences, St. Mary’s University Twickenham, London, UK; 3 Union Cycliste Internationale, Aigle, Switzerland; 4 Psychiatric University Hospital, University of Zurich, Zurich, Switzerland

**Keywords:** Elite-level road cycling, Sports psychiatry, Athlete mental health, Mental health literacy, Men’s cycling, Women’s cycling

## Abstract

**Background:**

Elite-level athletes, including professional road cyclists, may be vulnerable to psychiatric disorders, yet help-seeking and care pathways are often impeded by stigma and budgetary constraints. Although sports psychiatry is a growing subdiscipline, mental health initiatives in elite-level road cycling remain limited, possibly leaving coaches and non-athlete personnel ill-equipped to support affected athletes and staff. Consequently, understanding mental health literacy levels among these stakeholders could help identify knowledge gaps and shape future interventions.

**Methods:**

This study explored mental health literacy in non-athlete stakeholders (leadership, management, medical, support, and technical personnel) from men’s and women’s elite-level road cycling teams, including Union Cycliste Internationale WorldTeams, ProTeams, and national teams. An online survey collected self-reported demographic data, bespoke items on general mental health awareness, and responses to the validated 35-item Mental Health Literacy Scale (MHLS), which assesses six literacy dimensions.

**Results:**

*n* = 181 participants (75.1% male/24.3% female/0.6% undisclosed) reported lower mental health literacy (*M*: 122.80 ± 12.50) than sporting and general population benchmarks. While 97.2% acknowledged the importance of mental health for performance and wellbeing, approximately one-quarter (*n* = 47) felt inadequately informed, and nearly a third (*n* = 59) reported limited support in their teams. Variations were observed across demographic and role-related variables, warranting further investigation.

**Conclusions:**

Mental health literacy among non-athlete stakeholders in elite-level cycling appeared comparatively lower than in other sports, underpinned by knowledge gaps. However, participants consistently supported educational initiatives, potentially reinforcing the importance of tailored schemes to address existing shortcomings and promote healthier, more sustainable competitive environments.

**Supplementary Information:**

The online version contains supplementary material available at 10.1007/s44192-026-00483-8.

## Introduction

Despite typically exhibiting higher levels of physical fitness, elite-level athletes are just as susceptible to psychiatric disorders as the general population [1]. Researchers have also identified a proportionally high mental health burden among sporting coaches [2]. Concurrently, sports psychiatry has evolved as a psychiatric subdiscipline closely aligned with sports medicine, focusing on prevention programmes and mental health promotion in different competitive domains [3, 4]. Likewise, sports psychiatry accentuates the importance of embedding tailored interventions and targeted services for mental illnesses to serve the needs of elite-level athletes and other stakeholders [3, 4].

Specifically, in both men’s and women’s elite-level road cycling, a sizeable prevalence of anxiety, depression, eating disorders, addiction, and sleep–wake disorders has been noted [5–8]; recently, male and female cyclists have begun to discuss their experiences of psychiatric disorders in academic papers and popular media, with some taking temporary or indefinite career breaks owing to mental ill-health [5, 6, 9, 10]. Alongside personal consequences for the rider, psychiatric disorders can have wider implications for their teams due to the substantial resources necessary for developing and managing these athletes [5].

As in other sports, these psychopathological trends are shaped by distinctive socioenvironmental determinants, such as performance and body weight pressures, travel and fatigue, exposure to interpersonal and online abuse, and additional stressors [5–13]. Notwithstanding these challenges, psychiatric help-seeking is comparatively low in elite-level road cyclists, in part due to enduring stigma in the peloton and negative perceptions about psychopharmacotherapy, which have been influenced by past doping violations [5, 6, 11–13].

Globally, elite-level road racing is regulated by the governing body, the Union Cycliste Internationale (UCI), which is mandated to advance cycling in all its forms [14]. Under this directive, the UCI oversees the highest levels of competition (*i.e.*, the UCI WorldTeams and UCI ProTeams races in men’s cycling and UCI Women’s WorldTeams and UCI Women’s ProTeams in women’s cycling) [14]. Moreover, the UCI has previously provided robust recommendations for psychiatric screening for riders through the Sport Mental Health Recognition Tool 1 (SMHRT-1) [15]. In the 2025 season, when this study was conducted, there were 18 UCI WorldTeams and 17 UCI ProTeams for men’s cyclists, and 15 UCI Women’s WorldTeams and 7 UCI Women’s ProTeams [16].

Furthermore, the UCI and five Continental Confederations represent more than 200 national-level cycling federations [17]. These entities assume diverse responsibilities, including managing elite-level athletes during international road cycling events (*e.g.*, for the Olympics or the UCI Road World Championships) [18, 19]. Although some national cycling federations have begun to create and pioneer mental health initiatives, the broader policy landscape offers significant opportunities for investment and strategic prioritisation in this area [11].

In various contexts, sports psychiatrists have been integrated into the medical units of competitive teams to facilitate care pathways and the early detection of symptoms, such as in professional basketball and American football [20]. To the authors’ knowledge, the presence of sports psychiatrists is scarce in elite-level road cycling. That said, certain road cycling teams utilise sports psychology specialists, primarily for performance-based purposes; simultaneously, press reports indicate that these practitioners provide some forms of mental health support for male and female riders (*e.g.*, talking therapies) [21].

Equally, evidence suggests that coaches and support staff can influence athletes’ help-seeking behaviours, particularly by recognising symptoms and facilitating access to support, as well as shaping team environments and attitudes about mental health [22–25]. While this literature points towards a possible role for non-athlete personnel in supporting the wellbeing of athletes, the extent to which their mental health literacy directly influences athlete outcomes is yet to be firmly established across all sports.

Nevertheless, in lieu of accessible psychiatric expertise, and given their proximity to riders, it is conceivable that coaches and other stakeholders in road cycling organisations are working with athletes with undiagnosed and/or untreated mental health disorders, which might potentially exacerbate extant problems if mental health literacy is low. This was exemplified by the former rider and 2017 Giro d’Italia winner, Tom Dumoulin, when he took a competitive break due to mental ill-health and reflected that: “everyone saw me unhappy, *but no one could help me*” (italics ours) [9].

Analogous patterns have been identified in sports beyond cycling, placing an onus on non-athlete staff (especially coaches) to safeguard vulnerable competitors, for which they may not have received adequate training in psychiatric symptom recognition and appropriate referral mechanisms [22, 23]. In turn, the emergence of these responsibilities has illustrated a need for greater attention to the requisite skills and knowledge for lay stakeholders to respond to mental health concerns [23, 24]. This is important since concerns about team reactions to the disclosure of mental health issues could inhibit help-seeking in athletes [25]. However, presently, work exploring education for psychological trauma and post-traumatic stress in certified cycling coaches has indicated a notable absence of dedicated programmes addressing these topics [26].

Previous inquiries have examined mental health literacy among coaches and support staff across several sports and national settings, but comparable data in elite-level road cycling remains limited [27–29]. Thus, it is unclear to what extent team staff possess adequate knowledge and competencies to effectively engage with mental health issues that are arising within elite cycling environments.

Against this background, the current study is one of the first to explore the levels of mental health literacy in non-athlete staff in men’s and women’s road cycling teams. Given the mental health burden in professional cycling and the barriers to care throughout the sport, understanding these dynamics in the community would be useful for identifying gaps, countering stigma, and broadening access to mental health resources. Concomitantly, these insights could inform practical initiatives to strengthen support structures and create healthier, more sustainable competitive environments for elite-level riders and team personnel themselves [5, 6].

## Methods

### Study design

This study employed an exploratory, cross-sectional online survey to provide an initial descriptive assessment of mental health literacy among non-athlete stakeholders working in men’s and women’s elite-level road cycling teams (*i.e*., staff involved in leadership, management, medical, support, and technical domains). For the purposes of this research, elite-level cycling was defined as encompassing UCI WorldTeams, UCI ProTeams, and national teams, which represent the highest levels of competition where organisations typically have specialised medical, performance, and coaching personnel [16, 18, 19].

### Survey design and measures

The survey was structured into three primary sections, commencing with self-reported demographic questions. Specifically, these questions covered age categories, gender, country of work, educational background, current team role, duration of employment in a road cycling team, team level (*i.e*., WorldTeam, ProTeam, or national team), and any prior professional experience in cycling, including as a professional rider.

Subsequently, participants responded to seven bespoke items assessing their perceptions and experiences related to mental health. These explored self-reported awareness of mental health topics, beliefs about the importance of mental health in professional cycling, and the extent to which mental health could be openly discussed within the team. Additionally, personnel were asked about the accessibility of team mental health services, the availability of support for athletes transitioning out of the sport, and the perceived value of mental health training. They were also asked whether they had ever encountered a situation where a rider experienced a mental health issue but felt unsure about how to provide adequate assistance.

Finally, respondents completed the Mental Health Literacy Scale (MHLS), a validated 35-item measure [30]. The scale comprises six dimensions of mental health literacy, namely: “recognition of disorders” (items 1–8), “knowledge of risk factors and causes” (items 9 and 10), “self-treatment knowledge” (items 11 and 12), “knowledge of professional help available” (items 13–15), “knowledge of how to seek mental health information” (items 16–19), and “attitudes that promote recognition and appropriate help-seeking” (items 20–35) [30]. These are based on an established conceptual framework for mental health literacy [31]. Upon initial testing, the MHLS has demonstrated sufficient content and structural validity, as well as good test–retest reliability (*r* = 0.797, *p* < 0.001) and internal consistency (α = 0.87) [30].

Items 1–15 of the MHLS are scored on a four-point Likert scale, with response options ranging from “very unlikely” to “very likely” (items 1–10 and 13–15) and “very unhelpful” to “very helpful” (items 11–12). Items 10, 12, and 15 are reverse-scored. Furthermore, items 16–28 are scored on a five-point Likert scale with options ranging from “strongly agree” to “strongly disagree” and from “definitely unwilling” to “definitely willing” for items 29–35. The total score is derived by summing all item responses, and higher scores indicate higher levels of mental health literacy, with a maximum score of 160 [30].

Previously, studies have deployed the MHLS in various contexts, including in sports coaches in the United Kingdom and coaches and athletic trainers in Canada [27–29]. Accordingly, this enabled cross-comparisons between the current sample in cycling and past research on mental health literacy across disparate sporting settings.

### Procedure

The survey was hosted on Qualtrics and was available from October 2024 to March 2025. Prior to data collection, a review request was submitted to the Ethics Commission of the Canton of Bern (KEK Bern; Req-2024-00954). The Commission determined that the study falls outside the scope of the Swiss Human Research Act (HFG). The requirement for formal ethics approval was therefore waived under this legislation. The study involves survey-based data collection on attitudes and awareness among sports professionals and does not constitute human research as defined under the HFG, which governs clinical and biomedical research involving health-related personal data or biological material [32]. The study was conducted in accordance with the principles of the Declaration of Helsinki. Participants reviewed an information page before accessing the survey, and informed consent was obtained electronically from all individual participants.

Owing to the lack of centralised information on individuals working for elite-level road cycling teams, individuals and organisations involved in elite-level cycling (*e.g.*, journalists, UCI members, national federation representatives, and other professional contacts) were asked to share the survey with their networks to maximise participation. The survey was also promoted at cycling-specific and UCI events. To be eligible, respondents had to be aged 18 years or older and working for a WorldTeam, a ProTeam, or a national road cycling team in either men’s or women’s racing.

Participation was anonymous and voluntary, with respondents having the right to exit at any time without reason. That said, personnel were given the option to voluntarily provide their email address to enter a prize draw. One randomly selected participant received a 12-month subscription to Rouvy, a virtual cycling training platform. To maintain confidentiality, email addresses were stored separately from survey responses and deleted after the prize draw was completed.

### Data analysis

Survey data were exported from Qualtrics and uploaded to IBM SPSS Statistics (Version 26, IBM Corp.) for descriptive analysis. Calculations included statistical parameters, such as mean values, standard deviations, medians, quartiles, and value ranges. Normality was assessed using the Shapiro–Wilk test (*p* = 0.267). Despite no significant deviation from normality, non-parametric tests were employed as a conservative approach owing to the presence of small and unbalanced subgroup sizes. Subgroup comparisons were made using the Mann-Whitney U test. Percentage differences were assessed using the Chi^2^ test. Effect sizes for subgroup comparisons were calculated using Cohen’s d to estimate the magnitude of observed differences. A value of *p* < 0.05 was considered statistically significant.

Given the exploratory nature of the study and the number of subgroup comparisons conducted, no correction for multiple testing was applied; therefore, these initial findings should be interpreted as hypothesis-generating. The MHLS dimensions were compared using relative values, with the absolute values scaled to the achievable maximum for the respective dimension(s).

## Results

### Sample characteristics

One hundred and eighty-one members of elite-level road cycling teams completed the survey, out of four hundred and one individuals who initiated participation. Of these, 136 (75.1%) were male, 44 (24.3%) were female, and one respondent (0.6%) preferred not to disclose their gender. Respondents came from 28 countries, including 81 (44.3%) from Belgium, 20 (10.9%) from Netherlands, 16 (8.7%) from France, 14 (7.7%) from Spain, and 12 (6.6%) from Switzerland. The remaining countries are presented in the Supplementary Materials (S1).

Ages ranged throughout the sample, with 59 respondents aged 18–34 years (32.6%), 43 between 35–44 years (23.8%), 55 between 45–54 years (30.4%), and 24 aged 55 years or over (13.2%). 105 respondents (58.0%) were educated to university level, 53 (29.3%) had completed high school, and 23 (12.7%) reported vocational or other forms of education. 98 participants (54.1%) worked for WorldTour teams, 43 (23.8%) for ProTour teams, and 40 (22.1%) for national teams.

For team roles, the majority of participants worked in leadership positions (*e.g.*, sporting director, head coach, performance analyst) (60; 33.1%), followed by 39 (21.5%) medical staff (*e.g*., team doctor, psychologist, nutritionist), 36 (19.9%) in management roles (*e.g.*, general manager, logistics manager, press officer), 32 (17.7%) support personnel (*e.g.*, masseur), and 14 (7.7%) technical staff (*e.g.*, mechanic, equipment manager). Most individuals (67; 37.0%) had been involved in cycling for over 10 years, and 114 (63.0%) had previously competed professionally as a rider.

### General mental health items

Forty seven respondents (26.0%) “somewhat” or “strongly” disagreed that they were adequately informed about mental health, though 176 (97.2%) “somewhat” or “strongly” agreed that mental health is important for performance and wellbeing.

Moreover, 43 (23.8%) “somewhat” or “strongly” disagreed that people in their team were able to openly speak about mental health, and 59 (32.5%) “somewhat” or “strongly” disagreed that team members could access support (*e.g.*, talking to a psychiatrist). Eighty-two (45.3%) “somewhat” or “strongly” disagreed that their team provides mental health support for cyclists transitioning or preparing to retire.

Notably, 171 participants (94.5%) “somewhat” or “strongly” agreed that learning about mental health was valuable for coaches and team directors.

### Mental health literacy scale

#### Overall scores

The mean score on the MHLS was 122.80 across this sample, out of a maximum possible score of 160, as shown in Table 1.

**Table 1:** Mental health literacy scale overview

Role	*n*	Mean*	SD	MIN	Q1	Median	Q3	MAX
Management	36	125.0	12.0	96	117.0	123.0	133.0	148
Leadership	60	121.0	10.5	100	115.0	121.0	126.8	148
Medical	39	128.1	12.4	94	122.0	127.0	137.0	148
Technical	14	113.1	10.5	96	104.0	112.0	124.2	129
Support	32	121.4	14.5	93	110.2	117.5	134.0	152
Mental health literacy scale–total sample	181	122.80	12.50	93	122.0	122.0	132.0	152

Abbreviations: SD = standard deviation; MIN = minimum; MAX = maximum; Q1 = first quartile; Q3 = third quartile

^*^Statistically significant between-group differences (*p* < 0.05) are reported in the text

Exploratory subgroup analyses indicated some between-group differences across key demographic and role-related variables. Specifically, statistically significant differences were observed in team roles (*i.e.,* between management and technical personnel (*p* = 0.006; *d* = 1.02), leadership and medical staff (*p* = 0.001; *d* = 0.62), leadership and technical staff (*p* = 0.029; *d* = 0.75), and between medical and technical staff (*p* < 0.001; *d* = 1.25) and support staff (*p* = 0.022; *d* = 0.50)).

Correspondingly, statistically significant differences were also observed between respondents with lower-level secondary education and university education (*p* = 0.003; *d* = 1.07) and advanced university (*p* = 0.001; *d* = 1.18), as well as between high school and university education (*p* = 0.011; *d* = 0.46) and advanced university (*p* < 0.001; *d* = 0.68). Furthermore, personnel who identified as female had significantly higher MHLS scores than men (*p* = 0.003; *d* = 0.53). Interestingly, there were no statistically significant differences between team levels (*i.e.,* World Tour, Pro Tour, and national teams).

Overall, these variations were most consistently observed between technical personnel and other roles, as well as across educational and gender groupings. However, given the number of subgroup comparisons, the absence of correction for multiple testing, and the exploratory nature of the analyses, these findings should be interpreted with caution. Accordingly, the observed effect sizes should be considered indicative rather than definitive estimates of the magnitude of these differences.

#### Dimensional analyses

**Fig. 1:**
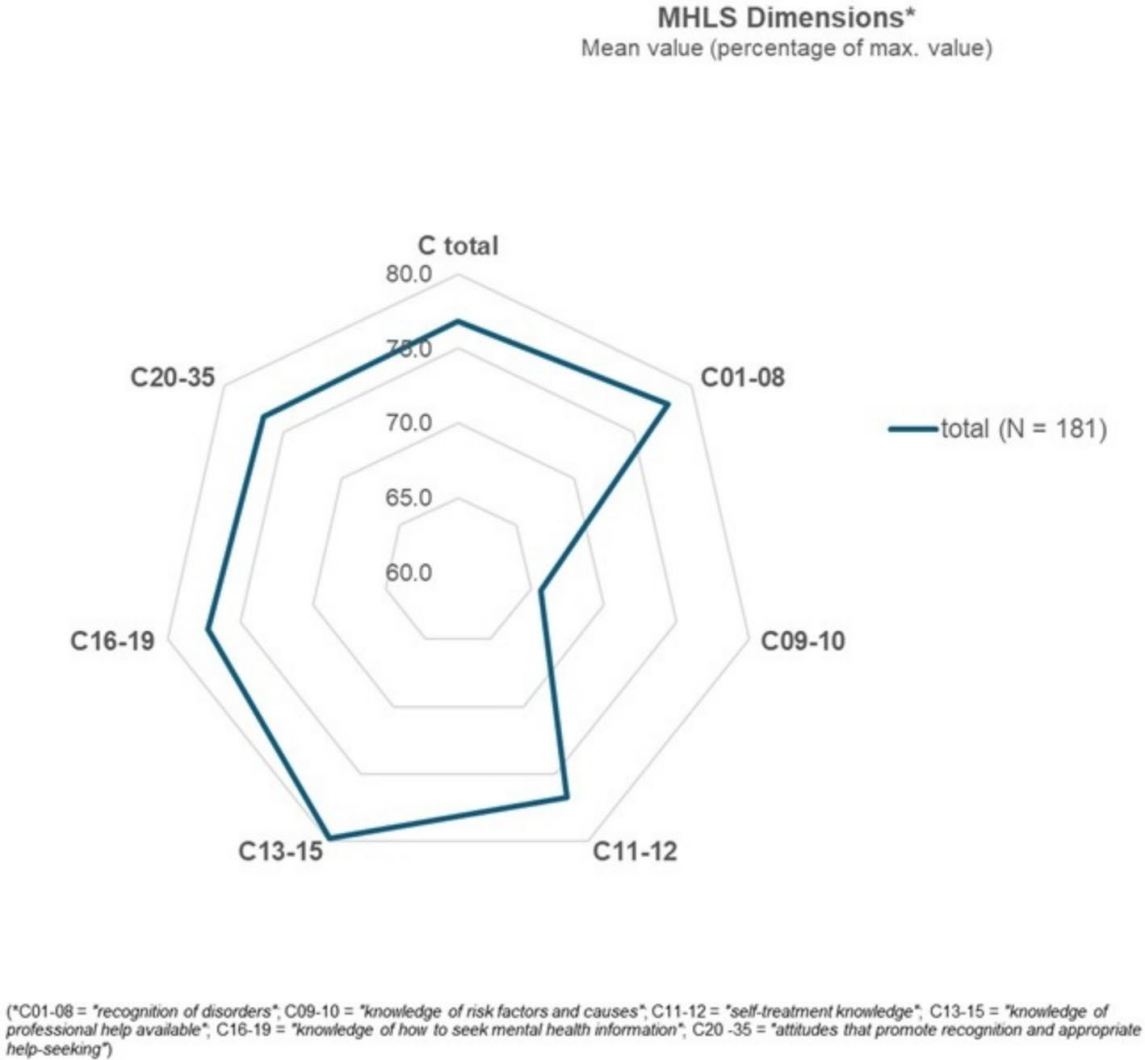
Mental Health Literacy Scale dimensions in the whole sample

Owing to the absence of cut-off thresholds for MHLS subscale interpretation, dimensional findings are presented as relative comparisons within the sample. Proportionally lower scores were observed for the “knowledge of risk factors and causes”, while higher scores were observed for “knowledge of professional help available” (Fig. 1).

**Fig. 2:**
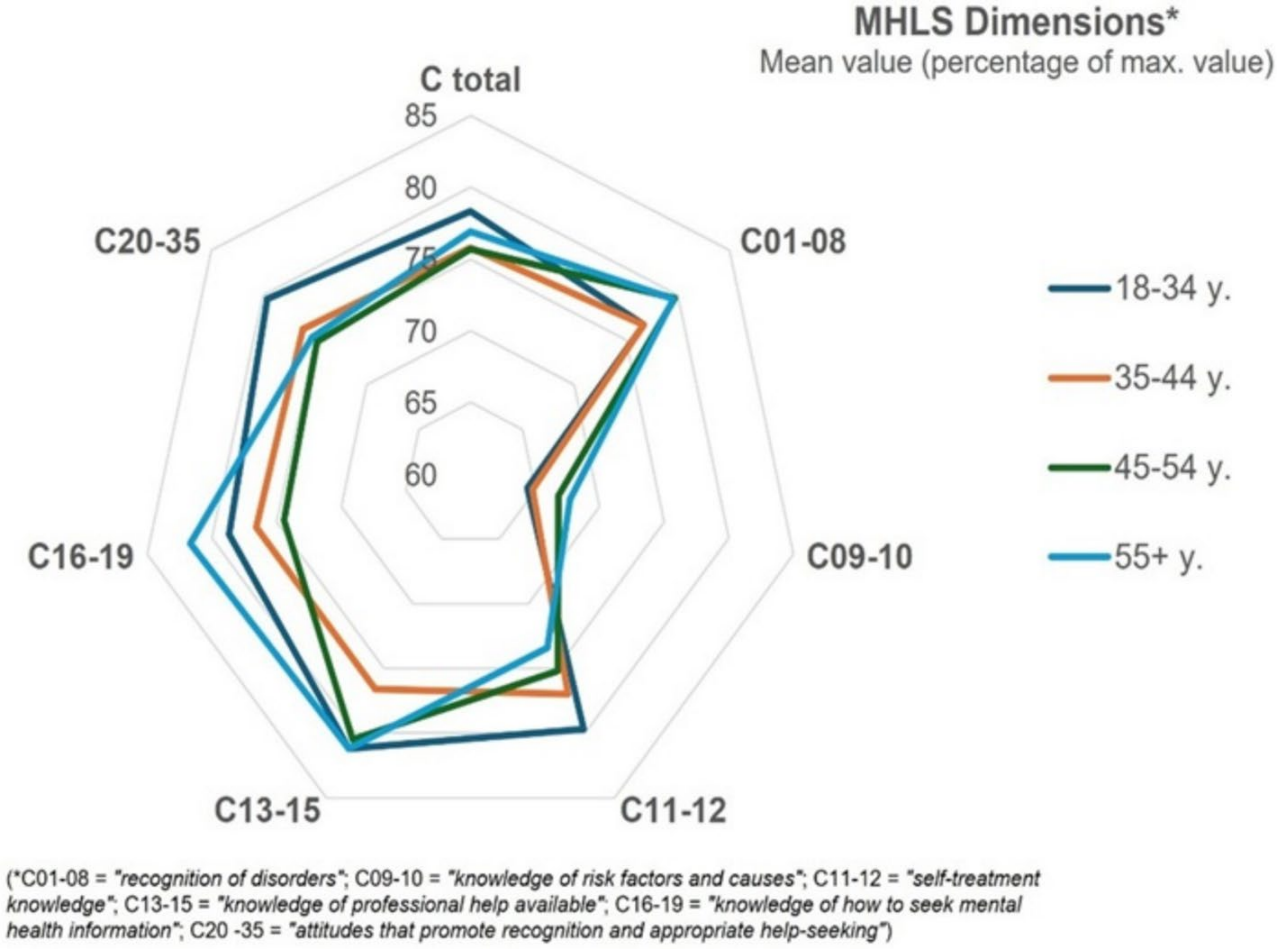
Mental Health Literacy Scale dimensions by age

**Fig. 3:**
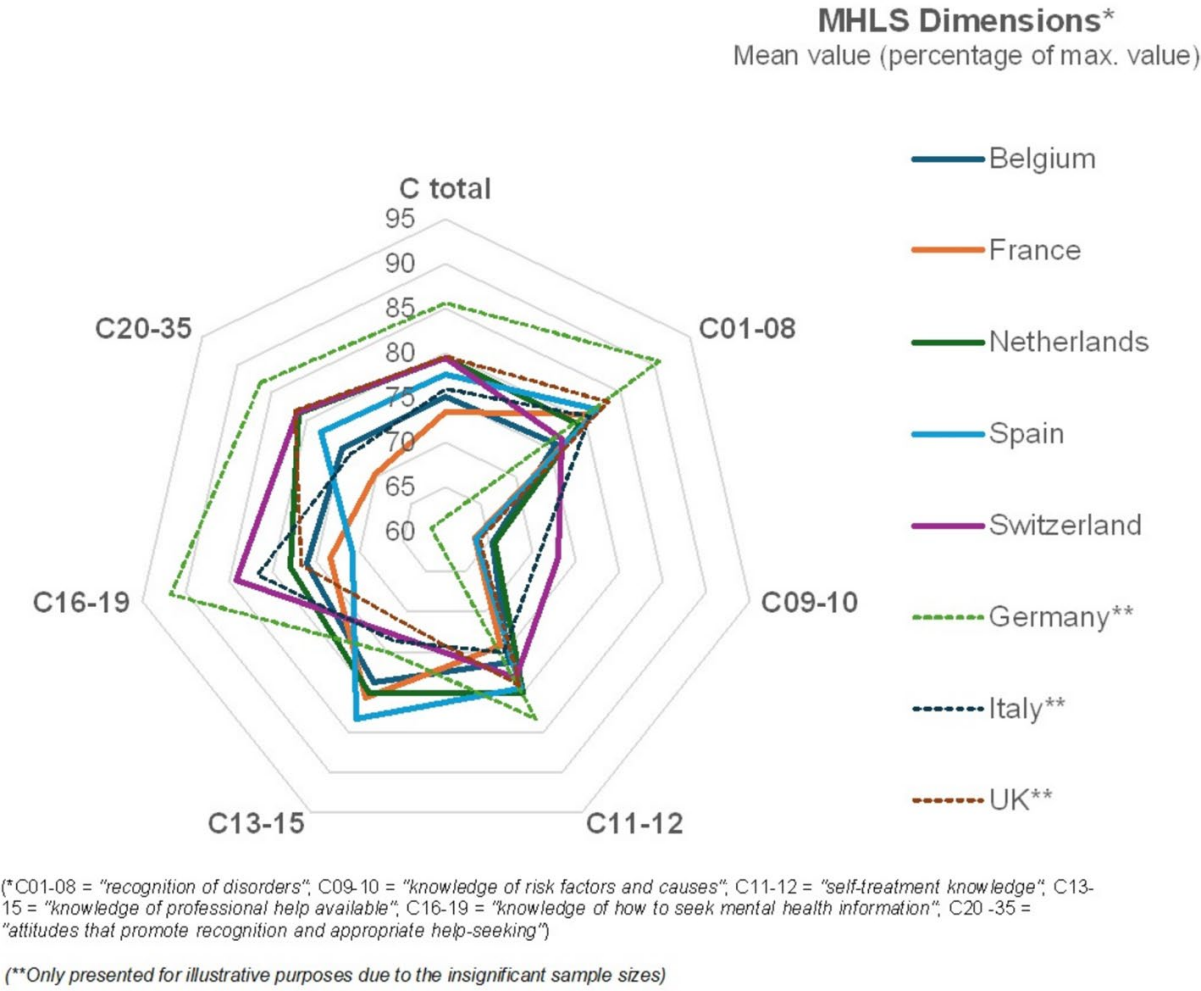
Mental Health Literacy Scale dimensions by country where the respondent was based (countries with the largest sample sizes shown; remaining countries not displayed for clarity)

Similar patterns of comparatively lower scores in the “knowledge of risk factors and causes” dimension were discernible by age and country of work groupings (Figs. 2 and 3). Notably, personnel from younger age groups (*i.e.*, (18–34 and 35–44)) had slightly elevated total scores and scored higher on the “self-treatment knowledge” and “attitudes that promote recognition and appropriate help-seeking” dimensions than older participants. Nonetheless, respondents aged 55 and older exhibited higher scores on “knowledge of how to seek mental health information” and “knowledge of risk factors and causes” than other age categories.

The lower scores for “knowledge of risk factors and causes” were also evident in the dimensional analyses by country of staff assignment. Here, respondents in Switzerland scored higher than the significant country samples overall, and especially for “knowledge of how to seek mental health information”. Equally, alongside stakeholders in the Netherlands, respondents in Switzerland scored higher on “attitudes that prompt recognition and appropriate help-seeking”. Elsewhere, participants from Spain achieved higher results on “knowledge of professional help available” and comparably higher scores on “recognition of disorders”.

All other dimensional analyses by subgroup and demographics (*i.e*., team role, team level, gender, education, and length of time on the team) are available in the Supplementary Materials (S2-S6).

## Discussion

This study is among the first to explore mental health literacy in non-athlete stakeholders working for men’s and women’s elite-level road cycling teams. In relation to relevant literature, international elite-level cycling team members appeared to demonstrate lower MHLS scores than participants from other sports, notwithstanding differences in sampling strategies, cultural contexts, participant characteristics, and methodological approaches [27–29]. For example, in two inquiries conducted in the United Kingdom, higher scores of 123.10 in 103 coaches and 129.5 in 184 athletic coaches, respectively, were identified (compared to 121.0 for road cycling team leadership in this survey) [27, 28]. In Canada, 80 coaches and athletic trainers had a higher average MHLS total of 131.48 [29]. Equally, the overall total for the non-athlete cycling personnel in the current analysis was slightly lower than a baseline (127.38) from the initial community sample of 372 individuals used to develop the MHLS [30].

In sum, when considered alongside existing relevant research (albeit with comparative caveats), these indicative disparities may suggest proportionally lower levels of mental health literacy in this sample from elite-level cycling. Notably, given the potential for self-selection bias in the design, it is possible that the observed mental health literacy levels also represent an overestimation of those present in the wider population of non-athlete personnel in cycling. 

The underlying reasons for these patterns go beyond the scope of this investigation, especially given its cross-sectional, exploratory nature, but are likely shaped by complex sociocultural, socioeconomic, and contextual determinants. Specifically, the higher MHLS scores registered by women in the sample are consistent with prior findings in sporting settings [29–31]. Across professional cycling, women are still underrepresented in senior decision-making positions, though recent progress has been made to redress gendered inequalities [6]. While structural factors exceed the scope of the current study, these traditional imbalances may hypothetically influence how mental health knowledge is translated into organisational practices [6]. However, this interpretation remains speculative and warrants further investigation.

Currently, mental health initiatives remain scarce within cycling, and this could also have affected the MHLS results [5, 6, 11–13]. Despite not using the MHLS, research into psychological trauma education among professional cycling coaches revealed a paucity of formal education on such topics, with similar shortcomings evident in inquiries into national federations [11, 26]. Additionally, unlike other sports, there are currently no requirements for cycling teams to provide standardised psychoeducation or embedded psychiatric services, although SMHRT-1 screening is strongly recommended by the UCI during routine medical examinations of riders [15]. Nevertheless, this might also explain the variable levels of literacy and reported availability of mental health care identified in the current findings, but again this relationship is not directly determinable in the current study.

As the sample comprised respondents from 28 countries, it is plausible that cultural heterogeneity in mental health literacy, stigma, and help-seeking limited these overall scores [33, 34]. Analogously, entrenched sociocultural beliefs have endured in road cycling, such as masculinised paradigms and an acceptance of pain and suffering as necessary components of competition [5, 6]. Adverse ideologies have been reflected in reports accentuating a “synonymous” relationship between cycling and pain [34], but recent media portrayals suggest gradual shifts towards more sensitivity about mental health from newer generations (*e.g.*, [5, 6, 9, 10, 35–38]). That said, these dynamics could inform the observed inter-age group dimensional differences, particularly since 63% of respondents had previously raced professionally, although this should be considered in light of the study design and sampling approach.

Importantly, the comparatively lower MHLS scores in elite-level road cycling teams may conceivably entail negative implications for help-seeking behaviours and early detection in both athletes and staff. For example, support and technical personnel frequently endure onerous travel schedules and high-pressure environments, especially in Grand Tours and multi-stage races, while receiving less financial compensation than athletes and other team stakeholders [39]. These conditions might correlate with heightened psychiatric vulnerabilities [2], but reduced access to training could restrict their exposure to mental health education, contributing to the diminished literacy scores in these roles. Notably, physiotherapists tend to develop close personal relationships with riders and can act as informal confidants when psychological issues are disclosed [40]. Simultaneously, their trusted position within team environments illustrates the value of equipping them with basic mental health resources to recognise and appropriately report early signs of distress.

Accordingly, these initial findings highlight substantial opportunities for schemes aimed at enhancing awareness and mental health literacy. In various sports, universal interventions (*e.g.*, workshops and training materials) have been implemented, resulting in positive changes in help-seeking attitudes and stigma [41–43]. With broader endorsement, tailored projects may represent a beneficial avenue in road cycling, due to the high rates of mental illness in the sport [5–7]. Encouragingly, 94.5% of this sample affirmed the potential benefits of education on mental health, perhaps signalling a larger acceptance in the community to engage with appropriate initiatives to improve mental health literacy.

To that end, training for stakeholders at organisational events or integrated into existing courses for team personnel could be considered as a possible strategy, focusing on the six dimensions of mental health literacy, although their effectiveness would require further evaluation [28]. The UCI already oversees mandatory training modules for sports directors, medical staff, and other team officials [44]. Consequently, reinforcing mental health content throughout these certifications (unrelated to performance-based aspects) may facilitate wide-reaching and standardised knowledge dissemination without requiring bespoke administrative structures. Sports psychiatrists could help refine and deliver these schemes to ensure alignment with evidence-based practices [3, 4, 20].

Likewise, as argued in previous work on athletic coaches [31], programmes targeted at enhancing specific dimensional knowledge in demographic subgroups (*e.g.*, men) and in distinct positions or national settings could be advantageous. Peer-led schemes have proven effective in enhancing mental health literacy among athletes [43]; they may be adapted and selectively trialled by cycling organisations. Yet, as existing evidence indicates, any initiative must be measurable and attuned to the nuances of cycling [43], thus requiring comprehensive knowledge of its sociocultural nuances, along with sufficient funding and stakeholder commitments.

## Strengths, limitations, and future research directions

This study provides preliminary evidence on the levels of mental health literacy in non-athlete personnel in men’s and women’s elite-level road cycling teams. Utilising the validated MHLS questionnaire enabled cross-comparisons with previous work, and our results showed that non-athlete stakeholders involved in cycling have proportionally lower MHLS scores than individuals in other sports and populations [27, 29–31]. The robust sample size is another key strength, as is the inclusion of diverse stakeholders across national contexts, team levels, and positions in professional cycling.

Nonetheless, the study has several limitations. Firstly, since recruitment relied on voluntary participation and dissemination through professional networks and industry-specific channels, individuals with greater interest or awareness of mental health may have been more likely to participate. Consequently, the sample may not be fully representative of all non-athlete personnel in elite-level road cycling. Indeed, this could have led to an overestimation of mental health literacy levels in this sample, and participation itself may reflect pre-existing attitudes towards mental health.

Although the sample comprised 28 jurisdictions, geographical representation was uneven, and countries in Eastern Europe, South America, and parts of Asia were underrepresented. This is notable in the context of the growing internationalisation of professional road cycling, evidenced by recent UCI investments to expand race calendars [45]. Equally, as mental health literacy and stigma can vary cross-culturally [33, 34], these limitations should be considered when interpreting the generalisability of the findings. Reliance on self-reported data may have introduced response bias, particularly social desirability or personal interpretations of mental health concepts. The cross-sectional design also only provides only a snapshot in time, limiting the ability to infer causality or track longitudinal changes.

Accordingly, future research should focus on longitudinal and follow-up designs to assess changes in mental health literacy following targeted or universal schemes, and consider extended sampling techniques to increase representation. Moreover, qualitative investigations would help capture perspectives and personal experiences from both athletes and staff, which in turn may be advantageous for developing relevant interventions. Finally, exploring the utility of peer-led support networks and reinforcing tailored mental health training within coaching certifications might yield practical solutions to bridge current gaps, but would require cycling-specific adaptations and robust monitoring and reporting.

## Conclusion

This study explored levels of mental health literacy in non-athlete personnel in men’s and women’s elite-level road cycling, suggesting that these may be slightly lower than those reported in other sports and values recorded within the general population. Differences across role and demographic variables were also observed, but these should of course be interpreted with appropriate caution given the exploratory and hypothesis-generating nature of the design.

Although sociocultural and socioenvironmental factors may influence existing knowledge gaps, these were not directly examined in the current analysis, thus providing an avenue for further investigation. Moreover, the cycling community’s willingness to engage with mental health education, as evidenced in this survey, as well as growing popular awareness about psychiatric issues, offers foundations for future research and targeted initiatives to improve conditions for athletes and staff. In turn, these efforts could support the evolution of healthier, more sustainable environments in elite-level cycling and across other sporting disciplines.

## Supplementary Information

Below is the link to the electronic supplementary material.

Supplementary file1 (DOCX 153 KB)

## Data Availability

All data generated or analysed during this study are included in this published article and its supplementary information files.

## Abbreviations

MHLS: Mental health literacy scale
SMHRT-1: Sport mental health recognition tool 1
UCI: Union cycliste Internationale
