# Supplementary material for: A cross-sectional study exploring mental health literacy among non-athlete personnel in elite-level road cycling
Source: Discov Ment Health. 2026 Jun 1;6(1):147. doi: 10.1007/s44192-026-00483-8 (PMC13438431; doi:10.1007/s44192-026-00483-8)
Supplement: Supplementary file 1 [file 44192_2026_483_MOESM1_ESM.docx]

**Supplemental Materials**

**S1. Participants by country**

| Country | *n* | % |
| --- | --- | --- |
| Andorra | 1 | 0.5% |
| Belgium | 81 | 44.3% |
| Brazil | 1 | 0.5% |
| Columbia | 1 | 0.5% |
| Croatia | 1 | 0.5% |
| Czech Republic | 2 | 1.1% |
| Denmark | 3 | 1.6% |
| Finland | 1 | 0.5% |
| France | 16 | 8.7% |
| Germany | 3 | 1.6% |
| Ireland | 3 | 1.6% |
| Italy | 7 | 3.8% |
| Luxembourg | 1 | 0.5% |
| Netherlands | 20 | 10.9% |
| Norway | 3 | 1.6% |
| Poland | 1 | 0.5% |
| Portugal | 1 | 0.5% |
| San Marino | 1 | 0.5% |
| Slovakia | 1 | 0.5% |
| Spain | 14 | 7.7% |
| Switzerland | 12 | 6.6% |
| UK | 9 | 4.9% |

**S2. Mental Health Literacy Scale dimensions by team level.**

(*C01-08 = *"recognition of disorders"*; C09-10 = *"knowledge of risk factors and causes"*; C11-12 = *"self-treatment knowledge"*; C13-15 = *"knowledge of professional help available"*; C16-19 = *"knowledge of how to seek mental health information"*; C20 -35 = *"attitudes that promote recognition and appropriate help-seeking"*)

MHLS Dimensions Mean value (percentage of max. value)

|  | World Tour | Pro Tour | Nat. Team |
| --- | --- | --- | --- |
| C total | 76.7 | 75.2 | 78.3 |
| C01-08 | 79.7 | 76.7 | 75.2 |
| C09-10 | 65.4 | 64 | 68.4 |
| C11-12 | 76.7 | 74.1 | 80.3 |
| C13-15 | 80.5 | 78.1 | 80.2 |
| C16-19 | 77.6 | 74.5 | 79.6 |
| C20-35 | 76 | 75.6 | 78.3 |

**S3. Mental Health Literacy Scale dimensions by gender.**

MHLS Dimensions Mean value (percentage of max. value)

|  | Male | Female |
| --- | --- | --- |
| C total | 75.8 | 79.8 |
| C01-08 | 77.6 | 79.3 |
| C09-10 | 64.7 | 69 |
| C11-12 | 76 | 79.5 |
| C13-15 | 80.1 | 79.2 |
| C16-19 | 76.1 | 81.4 |
| C20-35 | 75.4 | 80.8 |

**S4. Mental Health Literacy Scale dimensions by educational level.**

(*C01-08 = *"recognition of disorders"*; C09-10 = *"knowledge of risk factors and causes"*; C11-12 = *"self-treatment knowledge"*; C13-15 = *"knowledge of professional help available"*; C16-19 = *"knowledge of how to seek mental health information"*; C20 -35 = *"attitudes that promote recognition and appropriate help-seeking"*)

MHLS Dimensions Mean value (percentage of max. value)

|  | Lower level | High school | University | Advanced Uni | Other |
| --- | --- | --- | --- | --- | --- |
| C total | 69.9 | 74.3 | 77.5 | 79.4 | 79.7 |
| C01-08 | 72.2 | 76.7 | 78.3 | 78.9 | 82.8 |
| C09-10 | 61.1 | 64.6 | 67.7 | 64.8 | 66.1 |
| C11-12 | 70.8 | 73.3 | 78.4 | 80.9 | 75.9 |
| C13-15 | 72.2 | 78.3 | 81.2 | 81.1 | 81 |
| C16-19 | 72.8 | 74.9 | 77.3 | 81.7 | 77.1 |
| C20-35 | 68.6 | 73.5 | 77.6 | 80.1 | 80.6 |

**S5. Mental Health Literacy Scale dimensions by team role.**

(*C01-08 = *"recognition of disorders"*; C09-10 = *"knowledge of risk factors and causes"*; C11-12 = *"self-treatment knowledge"*; C13-15 = *"knowledge of professional help available"*; C16-19 = *"knowledge of how to seek mental health information"*; C20 -35 = *"attitudes that promote recognition and appropriate help-seeking"*)

MHLS Dimensions Mean value (percentage of max. value)

|  | Management | Leadership | Medical | Technical | Support |
| --- | --- | --- | --- | --- | --- |
| C total | 78.1 | 75.7 | 80.1 | 70.7 | 75.9 |
| C01-08 | 78.8 | 76.9 | 77.5 | 79.2 | 79.3 |
| C09-10 | 68.8 | 64.8 | 67.9 | 63.4 | 62.5 |
| C11-12 | 77.1 | 76.2 | 80.4 | 71.4 | 75.8 |
| C13-15 | 80.6 | 79.9 | 80.1 | 78 | 79.7 |
| C16-19 | 78.4 | 77.5 | 83.2 | 62.5 | 74.8 |
| C20-35 | 78.4 | 75.1 | 81.5 | 68.9 | 75.5 |

**S6. Mental Health Literacy Scale dimensions by length of time in a team.**

(*C01-08 = *"recognition of disorders"*; C09-10 = *"knowledge of risk factors and causes"*; C11-12 = *"self-treatment knowledge"*; C13-15 = *"knowledge of professional help available"*; C16-19 = *"knowledge of how to seek mental health information"*; C20 -35 = *"attitudes that promote recognition and appropriate help-seeking"*)

MHLS Dimensions Mean value (percentage of max. value)

|  | < 1 y. | 1-3 y. | 4-10 y. | > 10 y. |
| --- | --- | --- | --- | --- |
| C total | 77.1 | 78.2 | 74.3 | 81.7 |
| C01-08 | 74.5 | 77.8 | 77.5 | 79.2 |
| C09-10 | 65.4 | 63.8 | 65.4 | 67.4 |
| C11-12 | 82.7 | 75.6 | 78.3 | 75.3 |
| C13-15 | 81.4 | 80.6 | 79 | 80 |
| C16-19 | 84.2 | 77.6 | 79.6 | 73.7 |
| C20-35 | 84.4 | 77.6 | 79.4 | 72.3 |
